# Skull base reconstruction using *in situ* bone flap in patients with pituitary adenomas treated by endoscopic endonasal approach

**DOI:** 10.3389/fneur.2023.1194251

**Published:** 2023-06-14

**Authors:** Kaile Chen, Kexiang Dai, Zhiyuan Liu, Jinlai Liu, Kuo Yu, Ailin Lu, Peng Zhao

**Affiliations:** Department of Neurosurgery, First Affiliated Hospital of Nanjing Medical University, Nanjing, China

**Keywords:** pituitary adenoma, skull base reconstruction, *in situ* bone flap, post-operative CSF leakage, endoscopic endonasal approach, post-operative hospital stays

## Abstract

**Objective:**

The objective of this study is to study the effect of *in situ* bone flap (ISBF) repositioning, a recently proposed rigid skull base reconstruction technique, on patients diagnosed with pituitary adenoma undergoing endoscopic endonasal approach (EEA).

**Method:**

A retrospective analysis was conducted on 188 patients with pituitary adenomas who underwent EEA from February 2018 to September 2022. Patients were divided into the ISBF group and non-ISBF group, according to whether ISBF was used during skull base reconstruction.

**Results:**

Of the 75 patients in the non-ISBF group, 6 had postoperative cerebrospinal fluid (CSF) leakage (8%), while only 1 of 113 patients in the ISBF group (0.8%) had postoperative CSF leakage, indicating that the incidence of postoperative CSF leakage in the ISBF group was significantly lower than that in the non-ISBF group (*P* = 0.033). In addition, we also found that the postoperative hospitalization days of patients in the ISBF group (5.34 ± 1.24) were significantly less than those in the non-ISBF group (6.83 ± 1.91, *P* = 0.015).

**Conclusion:**

ISBF repositioning is a safe, effective, and convenient rigid skull base reconstruction method for patients with pituitary adenoma treated by EEA, which can significantly reduce the rate of postoperative CSF leakage and shorten postoperative hospital stays.

## Introduction

Endoscopic endonasal approach (EEA) has gained great popularity in the treatment of pituitary adenomas due to advances in anatomic understanding and surgical technology (1). The successful outcome of these procedures depends on two critical factors as follows: complete resection of the lesion and effective reconstruction of the skull base. Despite the numerous reconstruction techniques that have been proposed and implemented in recent years, postoperative CSF leakage remains a persistent problem (2–5). Therefore, there is still a need to explore alternative reconstruction methods to minimize postoperative CSF leakage and improve patients' recovery.

The technique of ISBF repositioning, characterized by the creation of a bone flap that can be subsequently repositioned and secured within its original location to repair a bony defect resulting from surgical procedures during EEA, has gained widespread acceptance since its inception (6). However, the impact of this skull base reconstruction technique on patients with pituitary adenomas remains unclear and requires further investigation.

Our study findings reveal that the utilization of ISBF repositioning for skull base reconstruction during EEA surgery in patients with pituitary adenomas is both safe and effective, providing a feasible and efficient approach.

## Methods

### Data collection

This study retrospectively analyzed the data of patients diagnosed with pituitary adenomas who underwent EEA surgery from February 2018 to September 2022. All patients enrolled in the study, both in the ISBF and non-ISBF groups, will have tumor tissue retained intraoperatively for pathologic examination, and the pathologic findings must reveal a pituitary adenoma. To be included in the ISBF group, certain conditions also had to be met, including (1) the patient must not have undergone prior endonasal endoscopic tumor surgery so that there is an intact skull base structure for harvesting the bone flap; (2) the sphenoid sinus must be well-pneumatized; (3) the tumor must not infiltrate the bone so that the risk of tumor recurrence associated with bone flap repositioning is minimized. These criteria were carefully considered to ensure comparability and validity of the study results. Inclusion criteria were met by 188 patients, 113 of whom underwent skull base reconstruction with the ISBF repositioning technique and were categorized as the ISBF group, while the remaining 75 served as the non-ISBF group. Demographic information, surgical details, lesion characteristics, and clinical outcomes were evaluated and reviewed.

### Surgical technique

Prior to surgery, patients undergo a series of routine examinations to determine any contraindications to surgery or anesthesia. These contraindications include hard tumor texture suggested by imaging, inflammation in the nasal cavity, dumbbell-shaped tumors, and giant or large aggressive pituitary tumors that grow into the side of sella turcica, suprasellar region, or frontal base (7). Among other procedures, magnetic resonance imaging (MRI) was performed to determine the anatomic relationship between the tumor and surrounding structures such as the optic nerve (ON), optic chiasm (OC), internal carotid artery (ICA), and cavernous sinus (CS) and to measure the size of the tumor. In addition, computed tomography (CT) imaging was routinely performed to assess any destruction of the sella bone and to evaluate the structure of the sphenoid sinus, particularly its bony components. All patients who can undergo surgery received prophylactic antibiotics (cefuroxime sodium) 30 min before surgery.

**Figure 1:**
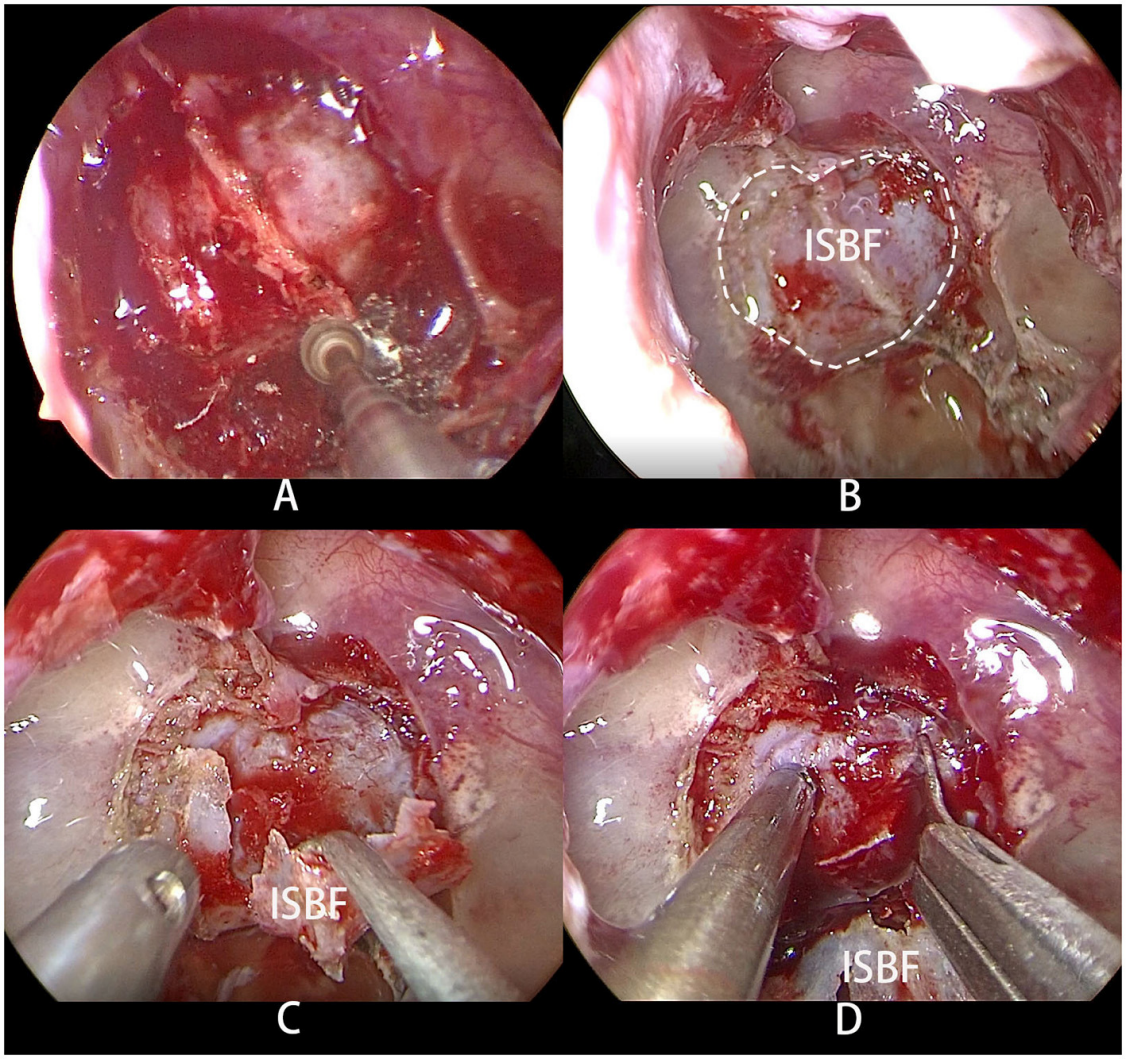
**(A)** Osteoplastic craniotomy was performed with a microdrill to obtain the bone flap. **(B)** The dotted line shows the range of ISBF. **(C)** The bone flap was folded to one side but not completely severed. **(D)** Upon incision of the dura mater, the pituitary adenoma is exposed.

The surgical procedures involved the use of the two-surgeon single or bi-nostril technique, which required a posterior septectomy to improve visualization and operation. After general anesthesia, patients were intubated and placed in the supine head position. The nasal cavities were packed with epinephrine-soaked cottonoids for several minutes to reduce mucosal bleeding. The anterior wall of the sphenoid sinus was opened wide with a microdrill to expose the sphenoid sinus. The *in situ* bone flap was harvested using a microdrill through an osteoplastic craniotomy, following a procedure previously described in the literature (6) (Figure 1). Visual gross total tumor resection was achieved in all patients in both groups.

After the removal of the pituitary adenoma, skull base reconstruction was performed. Different reconstruction methods were used for the ISBF and non-ISBF groups.

A multilayer closure technique was routinely employed in the non-ISBF group for skull base reconstruction. In this technique, the first layer was an artificial dura mater (ADM) filled into the tumor cavity as an inlay graft. The second layer was another ADM covering the incision as an onlay graft. The third layer comprised a polyurethane-based absorbable wound dressing (Nasopore^®^, Ethicon, Inc., United States), which was packed into the sphenoid sinus. In this process, fibrin glue was applied layer by layer to reinforce the structure and accelerate hemostasis. Additionally, the nasal cavity was packed with a polyvinyl formaldehyde medical sponge to provide extra support.

**Figure 2:**
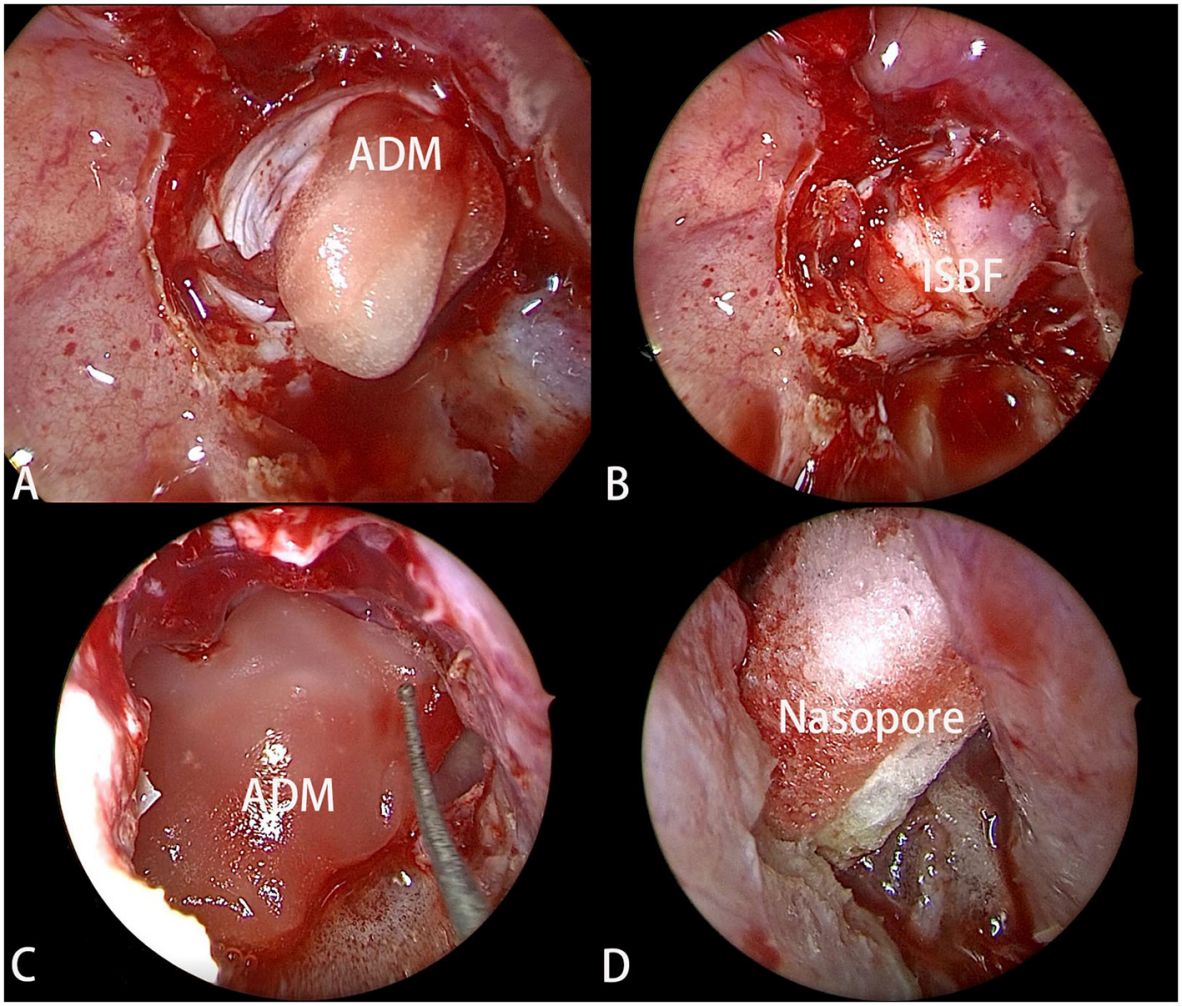
**(A)** The ADM (artificial dura mater) was filled into the tumor cavity as the first layer of reconstruction. **(B)** The ISBF was restored to the defect to complete the osseous reconstruction. **(C)** Another ADM was positioned over ISBF as the third layer. **(D)** The Nasopore was filled into as the last layer.

With the advent of the ISBF repositioning method (6), a different reconstruction strategy was employed for selected patients in the ISBF group. In this technique, an ISBF was used to repair the bone defect. Following the use of an ADM as the first layer, the previously obtained bone flap was repositioned as the second layer. Another ADM was placed over the bone flap as the third layer. Fibrin glue was routinely used during the procedure. The nasopore wound dressing was then introduced as subsequent layers (Figure 2), and the nasal cavity was packed with a polyvinyl formaldehyde medical sponge for added support.

In some cases with large tumor volumes, we used fat grafts from the thigh to fill the tumor cavity instead of ADM as the first-layer inlay graft in both the ISBF and non-ISBF groups. Our clinical practice did not involve the routine utilization of a pedicled nasoseptal flap (PNSF) for all patients, but rather reserved its application exclusively for situations characterized by a high-flow intraoperative CSF leak.

In our clinical practice, the adoption of prophylactic lumbar drainage in patients was not a customary procedure due to the limited body of evidence supporting its substantial impact on diminishing the incidence of postoperative cerebrospinal fluid (CSF) leakage (8–10). The surgical procedures were performed by a highly skilled and experienced surgeon (Dr. Peng Zhao) in the Department of Neurosurgery, First Affiliated Hospital of Nanjing Medical University. In this way, potential confounding factors arising from the learning curve effect were minimized, and the reliability and consistency of the surgical results were ensured (11).

## Diagnosis of post-operative CSF leakage

All patients were examined on CT on the same day shortly after the procedure and underwent MRI within 1–2 days after the procedure. All patients underwent a provocative “tilt test” to detect CSF rhinorrhea. In this test, patients tilt their heads downward with their noses in a dependent position for ~30 s. Repair failure with CSF rhinorrhea is typically indicated by a continuous watery discharge from one or both nostrils, while a thicker mucus-like discharge is not indicative of CSF rhinorrhea. In addition, we performed a glucose oxidation test on the nasal discharge of some patients, which can help in the diagnosis of CSF leakage. The beta2-transferrin test is widely considered the gold standard for the diagnosis of cerebrospinal fluid (CSF) leakage due to its exceptional accuracy and reliability (12). However, despite its diagnostic superiority, the test was not routinely prescribed in patients with suspected nasal CSF leakage in our institution due to several challenges. These challenges include the technical complexity of the test, the long test duration, and the high cost associated with performing the test (13).

### Statistical analysis

Comparison of categorical variables between the groups was performed using either the chi-square test or Fisher's exact test, depending on the sample size. Continuous variables were analyzed using a *t*-test for normally distributed data or a Wilcoxon rank sum test (Mann–Whitney U-test) for non-normally distributed data. All statistical analyses were performed using the Statistical Package for Social Sciences (SPSS Statistics, version 26, IBM Corp, Armonk, New York, USA), with a significance level of *P* < 0.05.

## Results

### Patient's basic characteristics

The mean age of patients in the ISBF group was 48.48 years, whereas the mean age of patients in the non-ISBF group was 49.87 years, with no statistically significant difference between the groups (*P* = 0.384). The distribution of female patients in both groups was comparable, with 62 (54.86%) women in the ISBF group and 45 (60%) women in the non-ISBF group (*P* = 0.486). Only one patient in the non-ISBF group had a history of previous EEA tumor surgery, whereas all patients in the ISBF group had no previous EEA tumor surgery, with no statistically significant difference between the two groups (*P* = 0.836). In the ISBF group, there were 55 functional tumors (0.49) and 58 non-functional tumors. Similarly, in the non-ISBF group, there were 30 functional tumors (0.4) and 45 non-functional tumors. Our statistical analysis revealed no significant difference between the two groups in terms of the proportion of functional tumors (*P* = 0.242). In addition, there were no statistically significant differences in demographic factors such as body mass index (BMI), history of radiation therapy, hypertension, and diabetes, which were previously found to be potential risk factors for postoperative CSF leakage (14–16) (Table 1).

**Table 1:** Patient's basic characteristics.

	**ISBF group**	**Non-ISBF group**	***P*-value**
No of patients	113	75	—
Age (Mean ± SD)	48.48 ± 14.40	49.87 ± 13.54	0.384
Sex			0.486
Male	51	30	
Female	62	45	
Hypertension			0.221
Yes	27	24	
No	86	51	
BMI (Mean ± SD)	24.74 ± 5.09 (kg/m^2^)	25.54 ± 3.57 (kg/m^2^)	0.333
Diabetes			0.091
Yes	8	11	
No	105	64	
Radiation therapy			-
Yes	0	0	
No	113	75	
Functional tumor			0.242
Yes	55	30	
No	58	45	
Previous EEA tumor surgery			0.863
Yes	0	1	
No	113	74	

### Surgical situation

The occurrence of intraoperative CSF leakage, which is known to be a high-risk factor for postoperative CSF leakage according to several studies, was not found to differ significantly between the two groups (*P* = 0.154) (14, 17). We reviewed the surgical records of all included patients and graded the intraoperative CSF leakage into four classes according to the criteria previously described: Grade 0, no CSF leakage observed confirmed by Valsalva maneuver; Grade 1, small “weeping” CSF leak confirmed by Valsalva maneuver without a visible diaphragmatic defect; Grade 2, moderate leak with definite diaphragmatic defect; and Grade 3: the presence of a large diaphragmatic and/or dural defect created during a suprasellar or transclival extended transsphenoidal approach (18). There was no statistically significant difference observed between the two groups, with respect to the distribution of intraoperative CSF grades (*P* = 0.158). We measured the size of the tumor by preoperative MRI and recorded the tumor size with the measured maximum diameter value of the tumor. No statistically significant difference was observed in tumor size between the two groups (*P* = 0.067), which remains a controversial factor in its influence on the incidence of postoperative CSF leakage, as reported by previous studies (14, 15, 19). Of the total number of patients, four in the ISBF group (0.035) and four in the non-ISBF group (0.053) received skull base reconstruction using fat grafts, and there was no significant difference in the application of fat grafts between the two groups (*P* = 0.820). There was no significant difference in the use of PNSF between the two groups (*P* = 0.719). Total visual resection of the tumor was achieved in all cases. There was no mortality in both groups (Table 2).

**Table 2:** Surgical situation.

	**ISBF group**	**Non-ISBF group**	***P*-value**
**Size of tumor**	18.78 ± 9.90 (mm)	23.45 ± 12.36 (mm)	0.067
**Intraoperative CSF leakage**			0.154
Yes	8	10	
No	105	65	
**Grade of intraoperative CSF leakage**			0.158
Grade 0 (No CSF leakage)	105	65	
Grade 1 (Small weeping CSF leakage)	6	8	
Grade 2 (Moderate CSF leakage)	2	2	
Grade 3 (large CSF leakage)	0	0	-
Operation time (Mean ± SD)	80.25 ± 29.35 (min)	108.73 ± 34.05 (min)	0.113
**Visual gross total resection**			-
Yes	113	75	
No	0	0	
**Use of fat graft**			0.820
Yes	4	4	
No	109	71	
**Use of PNSF**			0.719
Yes	1	2	
No	112	73	

### Clinical outcomes

Our study found that the use of ISBF repositioning during EEA surgery in patients with pituitary adenoma was associated with a significantly lower incidence of postoperative CSF leakage than in patients who did not undergo ISBF repositioning (*P* = 0.033). In addition, patients in the ISBF group had a significantly shorter mean hospital stay than patients in the non-ISBF group (*P* = 0.015). Further analysis revealed that there was no statistically significant difference between the two groups in terms of operative time or other postoperative complications such as epistaxis, meningitis, polyuria, or fever (*P* > 0.05). All patients with postoperative CSF leakage in both groups were successfully treated and recovered (Table 3).

**Table 3:** Clinical outcomes.

	**ISBF group**	**Non-ISBF group**	***P*-value**
**Post-operative CSF leakage**			**0.033**
Yes	1	6	
No	112	69	
**Post-operative hospital stays (Mean** **±SD)**	5.43 ± 1.24 (day)	6.83 ± 1.91 (day)	**0.015**
**Post-operative epistaxis**			0.258
Yes	4	0	
No	109	75	
**Post-operative meningitis**			0.163
Yes	1	4	
No	112	71	
**Post-operative polyuria**			0.329
Yes	10	10	
No	103	65	
**Post-operative fever**			0.183
Yes	3	6	
No	110	69	

## Discussions

Pituitary adenomas are the most common etiology of benign sellar masses, accounting for up to 10–15% of all diagnosed intracranial tumors, although there are other neoplastic, infectious, inflammatory, development, and vascular etiologies to be considered (20–22). Transsphenoidal surgery with an aim to achieve complete tumor resection is considered the first-line treatment for pituitary adenomas (23). With the development of medical technology, the EEA has become the predominant method for the removal of pituitary adenoma for its advantages of favorable cosmetic outcome, improved operative visualization, reduced invasiveness, quicker recovery, and higher gross total resection rate (24–28). Reconstruction of complex anterior skull base defects after EEA remains a challenge, despite neurosurgeons and otolaryngologists having explored various reconstruction materials in the past few decades, such as free autografts, intranasal vascularized flaps, synthetic dural replacement grafts, and pedicled nasoseptal flaps, either alone or in combination (2). Applying these conventional construction materials fails to reestablish bone defects rigidly. The purposes of skull base reconstruction, comparable to those of conventional transcranial procedures, are to establish watertight closure, eliminate dead space, repair the barrier between the intracranial cavity and the extracranial space, support the intracranial components adequately, and restore the original anatomical structure (29), which means rigid reconstruction may be essential. However, previous scholars generally argue that the utility of rigid materials such as cartilage or bone, absorbable or non-absorbable plates, and titanium mesh in skull base reconstruction is unnecessary and not recommended due to the higher risk of migration, infection, and injury to the neurovascular structure (18, 30, 31).

Now, a growing number of neurosurgeons hold more positive opinions about the rigid reconstruction of the skull base. They argue that rigid reconstruction is necessary and possibly effective in preventing acute or chronic headaches, pseudo meningocele, and non-specific discomfort caused by the bone defect, leading to better outcomes for patients (6). ISBF, as a recently proposed reconstruction material, has the advantages of convenient harvesting procedure, better biological compatibility, geometry matching that of bone defect, proximity to the complex initial anatomical structure of the skull base, stable fixation, and rapid healing of bone defect (32), which may avoid the risks mentioned above and become one of the most suitable materials for rigid reconstruction. In our study, the rate of postoperative meningitis in the ISBF group is lower than that in the non-ISBF group, although there was no statistical significance between the two groups (*P* = 0.163).

Based on our study, we found that the average operation time was shorter in the ISBF group (80.25 ± 29.35 min) than in the non-ISBF group (108.73 ± 34.05 min), which may appear counterintuitive since one would expect ISBF to be more complex and time-consuming. We found no statistically significant difference between the two groups (*P* = 0.113). During the surgical procedure, we discovered that obtaining and repositioning the bone flap was a straightforward process, contrary to our initial expectations. The bone flap was obtained incidentally during the opening of the posterior wall of the sphenoid sinus's opening, which was performed in both groups. In the ISBF group, the bone flap was not required to be removed through the nasal cavity and could be flipped to one side, as shown in Figure 1C. In contrast, in the non-ISBF group, the bone flap had to be removed through the nasal cavity, which sometimes required splitting the flap into multiple pieces if the nasal passage was too narrow, which, in turn, possibly increased the surgical time. Repositioning the flap was a quick process, taking only a few minutes for an experienced surgeon. It was also observed that the flap fit well into the defect and the complex anatomy of the skull base. However, we acknowledge that we cannot completely rule out the influence of the learning curve on the results, and future studies with newer patients will reduce this influence. The shorter operative time in the ISBF group, to some extent, implies that the *in situ* bone flap reduction technique is feasible and convenient.

Previous studies have shown that the application of ISBF can significantly reduce the rate of postoperative CSF leakage following EEA (30, 32, 33); however, few of them have focused on pituitary adenoma. Pituitary adenomas are the most common type of pituitary disorder. Therefore, it is influential to study the effect of ISBF repositioning on patients with pituitary adenoma. At our institution, the method of ISBF closure is routinely used for pituitary adenoma patients treated by the EEA surgery, with good results.

Diabetes insipidus (DI) is a prevalent complication following pituitary surgery, characterized by excessive urination. Postoperative DI can manifest as transient or permanent, with the latter being more frequent (34, 35). Scholars have identified a number of predictors of postoperative DI in their studies, such as younger age, larger tumor size, gross total surgical resection, and diaphragm opening during pituitary resection (36, 37). A recent investigation has revealed that the change in cephalocaudal tumor cavity diameter (CTCD) before and after surgery on enhanced coronal sections of MRI, which reflect the subsidence of the diaphragma sellae, is an independent predictor of postoperative DI onset in patients with pituitary adenoma (38). Moreover, a higher rate of postoperative DI is associated with a greater change in CTCD measured on coronal sections of MRI. It is postulated that narrowing the change in CTCD during the procedure could potentially decrease the incidence of post-operative DI.

**Figure 3:**
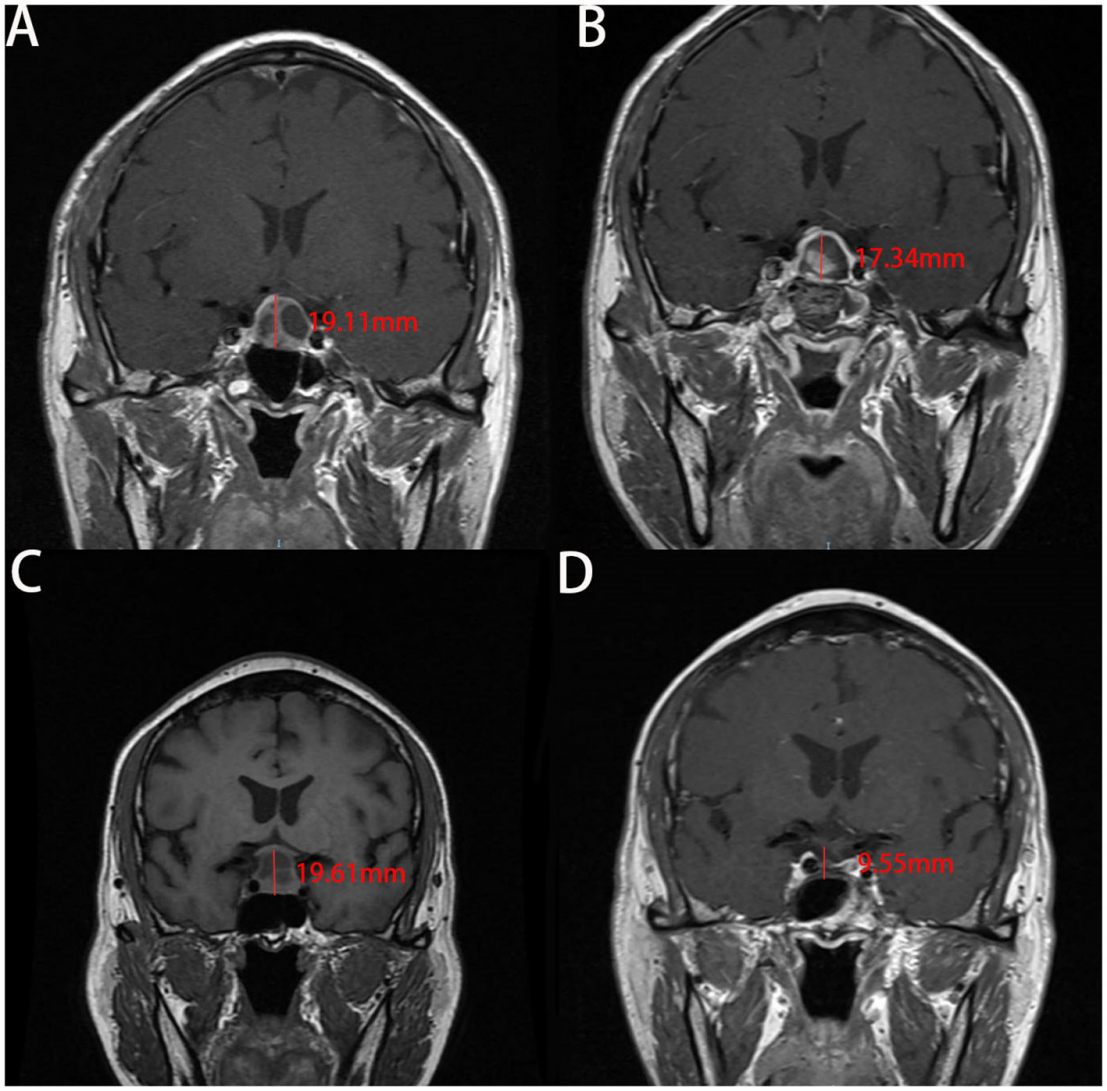
Images show the change of the cephalocaudal tumor cavity diameter (CTCD) after EEA measured on enhanced coronal sections of MRI in two cases. A and B are from the same patient in the ISBF group, while C and D are from the same patient in the non-ISBF group. **(A, B)** Before the surgery, the CTCD is **19.11** mm **(A)**. After surgery, the CTCD is **17.34** mm **(B)**. The change in CTCD measured on **coronal sections** of MRI is **1.77** mm. **(C, D)** Before the surgery, the CTCD measured on **coronal sections** of MRI is **19.61** mm **(C)**. After the surgery, the CTCD is **9.55** mm **(D)**. The change in CTCD is **10.06** mm.

**Figure 4:**
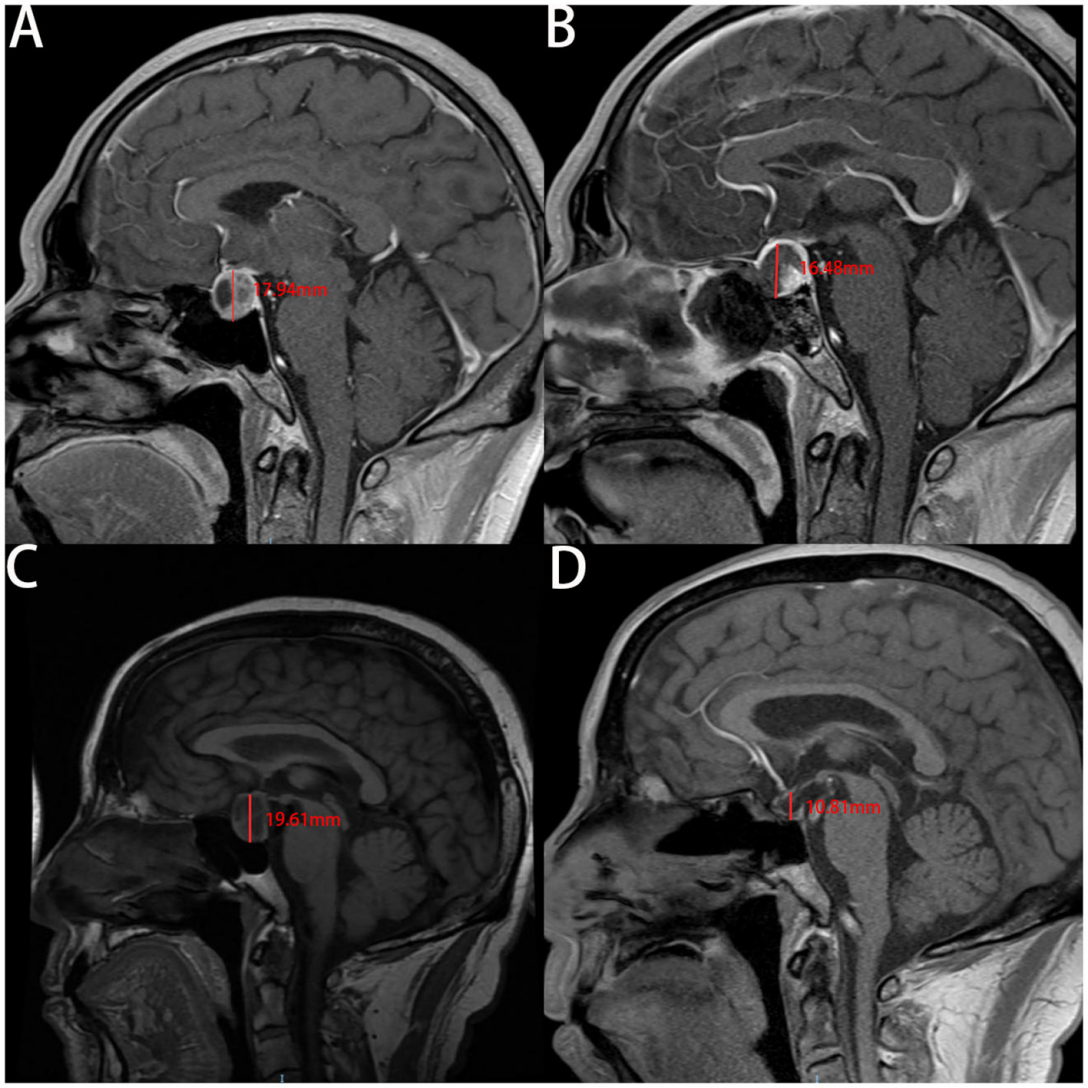
The images show the change of the cephalocaudal tumor cavity diameter (CTCD) after EEA measured on the enhanced **sagittal plane** of MRI in two cases. A and B are are from the same patient in the ISBF group, while C and D are from the same patient in the non-ISBF group. **(A, B)** Before the surgery, the CTCD is **17.94** mm **(A)**. After surgery, the CTCD is **16.48** mm **(B)**. The change in CTCD measured by the **sagittal plane** of MRI is **1.46** mm. **(C, D)** Before the surgery, the CTCD is **19.61** mm **(C)**. After the surgery, the CTCD is **10.81** mm **(D)**. The change in CTCD measured by the **sagittal plane** of MRI is **8.8** mm.

Our analysis of the patients' MRI scans revealed that the change in CTCD was higher in the non-ISBF group compared with the ISBF group (Figures 3, 4), potentially due to the absence of rigid skull base reconstruction. Additionally, although not reaching the statistical significance (*p* = 0.329), the incidence of postoperative polyuria was lower in the ISBF group (0.097) compared with the non-ISBF group (0.154), possibly due to the different skull base reconstruction methods employed.

We hypothesize that the increased diaphragma sellae subsidence and postoperative changes in CTCD may lead to a lower pituitary gland position and more severe traction on the pituitary stalk, resulting in greater injury to the hypothalamic-neurohypophysis system and a higher incidence of postoperative diabetes insipidus (DI). *In-situ* bone flap repositioning may be effective in preventing postoperative DI by reducing the degree of diaphragma sellae subsidence and change in CTCD after surgery. However, we were unable to confirm this hypothesis rigorously due to the lack of data, and further studies are necessary to substantiate this claim.

The ISBF repositioning technique has some limitations. First, when harvesting the bone flap, the surgeon must be careful to maintain the flap intact to prevent the migration of bone flap fragments and damage to the surrounding structure. This procedure may require more delicate surgical instruments and could take a young surgeon longer time to perform. In addition, our results show that the incidence of postoperative epistaxis was slightly higher in the ISBF group (0.037) compared with the non-ISBF group (0.000), although the difference was not statistically significant (*p* = 0.258). Bleeding from postoperative epistaxis was usually minor and could be managed with careful nasal packing, but it may also cause anxiety in patients. However, the clinical significance of this difference is unclear. Future studies may explore ways to further refine the ISBF technique.

However, our study has some limitations. Our study has all the shortcomings associated with retrospective studies. The sample size is still relatively small and not clearly randomized. There is a relatively large gap between the sample size of the ISBF group and the non-ISBF group. The data are from a single institution. We rely to some extent on the patient's complaints in assessing whether the patient can be discharged, so the criteria for assessing whether the patient can be discharged are not entirely objective. We acknowledge that although we attempted to minimize this bias through rigorous patient selection and matching procedures, the inclusion criteria for the ISBF group in our study may have resulted in fewer invasive tumors being selected in the ISBF group than in the non-ISBF group, potentially biasing the study results. Therefore, further prospective multi-institutional studies are required to confirm the utility of ISBF repositioning in pituitary adenoma patients undergoing EEA surgery.

## Conclusion

The ISBF repositioning is a reliable, convenient, and feasible skull base reconstructive technique for pituitary adenoma patients undergoing EEA surgery. Our preliminary results suggest that the ISBF combined with the multilayer closure technique can significantly lower the incidence of postoperative CSF leakage and shorten the hospital stays for patients.

## Data availability statement

The raw data supporting the conclusions of this article will be made available by the authors, without undue reservation.

## Ethics statement

The studies involving human participants were reviewed and approved by Ethics Committee of the First Affiliated Hospital of Nanjing Medical University. The patients/participants provided their written informed consent to participate in this study.

## Author contributions

PZ and KC conceived the idea and designed the study. PZ, KD, and JL performed the surgery. KC, KD, ZL, and KY are responsible for data collection and analysis. KC, KD, and PZ drafted the manuscript. KC, ZL, AL, and JL critically revised the manuscript for important intellectual content. PZ affirms that each listed author fulfills the established authorship criteria, and no eligible individuals meeting these criteria have been unintentionally excluded. All authors contributed to the article and approved the submitted version.

## References

[1] Castelnuovo P Dallan I Battaglia P Bignami M. Endoscopic endonasal skull base surgery: past, present and future. Eur Arch Oto-Rhino-Laryngol J Eur Arch Oto-Rhino-Laryngol (EUFOS): Ger Soc Oto-Rhino-Laryngol Head Neck Surg. (2010) 267:649–63. 10.1007/s00405-009-1196-0 20063006

[2] Sigler AC D'Anza B Lobo BC Woodard TD Recinos PF Sindwani R. Endoscopic skull base reconstruction: an evolution of materials and methods. Otolaryngol Clin North Am. (2017) 50:643–53. 10.1016/j.otc.2017.01.015 28372814

[3] Simal-Julian JA Pérez de San Román-Mena L Sanchis-Martín MR Quiroz-Tejada A Miranda-Lloret P Botella-Asunción C. Septal rhinopharyngeal flap: a novel technique for skull base reconstruction after endoscopic endonasal clivectomies. J Neurosurg. (2021) 3:1–6. 10.3171/2021.6.JNS203882 34678774

[4] Moon JH Kim EH Kim SH. Various modifications of a vascularized nasoseptal flap for repair of extensive skull base dural defects. J Neurosurg. (2019) 132:371–9. 10.3171/2018.10.JNS181556 30738381

[5] Harvey RJ Parmar P Sacks R Zanation AM. Endoscopic skull base reconstruction of large dural defects: a systematic review of published evidence. Laryngoscope. (2012) 122:452–9. 10.1002/lary.22475 22253060

[6] Jin B Wang XS Huo G Mou JM Yang G. Reconstruction of skull base bone defects using an *in situ* bone flap after endoscopic endonasal transplanum-transtuberculum approaches. Eur Arch Oto-Rhino-Laryngol J Eur Arch Oto-Rhino-Laryngol (EUFOS): Ger Soc Oto-Rhino-Laryngol Head Neck Surg. (2020) 277:2071–80. 10.1007/s00405-020-05911-1 32180016

[7] Shou XF Li SQ Wang YF Zhao Y Jia PF Zhou LF. Treatment of pituitary adenomas with a transsphenoidal approach. Neurosurgery. (2005) 56:249–56. 10.1227/01.NEU.0000147976.06937.1D 15670373

[8] Ahmed OH Marcus S Tauber JR Wang B Fang Y Lebowitz RA. Efficacy of perioperative lumbar drainage following endonasal endoscopic cerebrospinal fluid leak repair. Oto-Rhino-Laryngol Head Neck Surg. (2017) 156:52–60. 10.1177/0194599816670370 27677601

[9] D'Anza B Tien D Stokken JK Recinos PF Woodard TR Sindwani R. Role of lumbar drains in contemporary endonasal skull base surgery: meta-analysis and systematic review. Am J Rhinol Allergy. (2016) 30:430–5. 10.2500/ajra.2016.30.4377 28124655

[10] Cohen S Jones SH Dhandapani S Negm HM Anand VK Schwartz TH. Lumbar drains decrease the risk of postoperative cerebrospinal fluid leak following endonasal endoscopic surgery for suprasellar meningiomas in patients with high body mass index. Operative Neurosurg. (2018) 14:66–71. 10.1093/ons/opx070 29253284

[11] Park W Nam DH Kong DS Lee KE Park SI Kim HY. Learning curve and technical nuances of endoscopic skull base reconstruction with nasoseptal flap to control high-flow cerebrospinal fluid leakage: reconstruction after endoscopic skull base surgery other than pituitary surgery. Eur Arch Oto-Rhino-Laryngol J Eur Arch Oto-Rhino-Laryngol (EUFOS): Ger Soc Oto-Rhino-Laryngol Head Neck Surg. (2022) 279:1335–40. 10.1007/s00405-021-06877-4 34028580

[12] Abuabara A. Cerebrospinal fluid rhinorrhoea: diagnosis and management. Med Oral Patol Oral Cir Bucal. (2007) 12:E397–400. 17767107

[13] Mantur M Lukaszewicz-Zajac M Mroczko B Kulakowska A Ganslandt O Kemona H. Cerebrospinal fluid leakage—reliable diagnostic methods. Clin Chim Acta. (2011) 412:837–40. 10.1016/j.cca.2011.02.017 21334321

[14] Zhou Z Zuo F Chen X Zhao Q Luo M Jiang X. Risk factors for postoperative cerebrospinal fluid leakage after transsphenoidal surgery for pituitary adenoma: a meta-analysis and systematic review. BMC Neurol. (2021) 21:417. 10.1186/s12883-021-02440-0 34706659 PMC8555154

[15] Kim JS Hong SD. Risk factors for postoperative CSF leakage after endonasal endoscopic skull base surgery: a meta-analysis and systematic review. Rhinology. (2021) 59:10–20. 10.4193/rhin20.145 32785296

[16] Hutter G von Felten S Sailer MH Schulz M Mariani L. Risk factors for postoperative CSF leakage after elective craniotomy and the efficacy of fleece-bound tissue sealing against dural suturing alone: a randomized controlled trial. J Neurosurg. (2014) 121:735–44. 10.3171/2014.6.JNS131917 25036199

[17] Ivan ME Iorgulescu JB El-Sayed I McDermott MW Parsa AT Pletcher SD. Risk factors for postoperative cerebrospinal fluid leak and meningitis after expanded endoscopic endonasal surgery. J Clin Neurosci J Neurosurg Soc Aust. (2015) 22:48–54. 10.1016/j.jocn.2014.08.009 25439754

[18] Esposito F Dusick JR Fatemi N Kelly DF. Graded repair of cranial base defects and cerebrospinal fluid leaks in transsphenoidal surgery. Operat Neurosurg. (2007) 60(4 Suppl 2):295–303. 10.1227/01.NEU.0000255354.64077.66 17415166

[19] Jakimovski D Bonci G Attia M Shao H Hofstetter C Tsiouris AJ. Incidence and significance of intraoperative cerebrospinal fluid leak in endoscopic pituitary surgery using intrathecal fluorescein. World Neurosurg. (2014) 82:e513–23. 10.1016/j.wneu.2013.06.005 23811068

[20] Aldahmani KM Sreedharan J Ismail MM Philip J Nair SC Alfelasi M. Prevalence and characteristics of sellar masses in the city of Al Ain, United Arab Emirates: 2010 to 2016. Ann Saudi Med. (2020) 40:105–12. 10.5144/0256-4947.2020.105 32241168 PMC7118230

[21] Famini P Maya MM Melmed S. Pituitary magnetic resonance imaging for sellar and parasellar masses: ten-year experience in 2598 patients. J Clin Endocrinol Metab. (2011) 96:1633–41. 10.1210/jc.2011-0168 21470998 PMC3100749

[22] Lake MG Krook LS Cruz SV. Pituitary adenomas: an overview. Am Fam Physician. (2013) 88:319–27. 24010395

[23] Molitch ME. Diagnosis and treatment of pituitary adenomas: a review. Jama. (2017) 317:516–24. 10.1001/jama.2016.19699 28170483

[24] Wood JW Eloy JA Vivero RJ Sargi Z Civantos FJ Weed DT. Efficacy of transnasal endoscopic resection for malignant anterior skull-base tumors. Int Forum Allergy Rhinol. (2012) 2:487–95. 10.1002/alr.21062 22777956

[25] Eloy JA Vivero RJ Hoang K Civantos FJ Weed DT Morcos JJ. Comparison of transnasal endoscopic and open craniofacial resection for malignant tumors of the anterior skull base. Laryngoscope. (2009) 119:834–40. 10.1002/lary.20186 19296496

[26] Catapano D Sloffer CA Frank G Pasquini E D'Angelo VA Lanzino G. Comparison between the microscope and endoscope in the direct endonasal extended transsphenoidal approach: anatomical study. J Neurosurg. (2006) 104:419–25. 10.3171/jns.2006.104.3.419 16572655

[27] Rutland JW Goldrich D Loewenstern J Banihashemi A Shuman W Sharma S. The role of advanced endoscopic resection of diverse skull base malignancies: technological analysis during an 8-year single institutional experience. J Neurol Surg Part B, Skull Base. (2021) 82:417–24. 10.1055/s-0040-1714115 35573925 PMC9100431

[28] Bajaj J Chandra PS. Recent developments in endoscopic endonasal approach for pituitary adenomas. Neurol India. (2020) 68(Supplement):S79–84. 10.4103/0028-3886.287671 32611896

[29] Garcia-Navarro V Anand VK Schwartz TH. Gasket seal closure for extended endonasal endoscopic skull base surgery: efficacy in a large case series. World Neurosurg. (2013) 80:563–8. 10.1016/j.wneu.2011.08.034 22120292

[30] Snyderman CH Wang EW Zenonos GA Gardner PA. Reconstruction after endoscopic surgery for skull base malignancies. J Neurooncol. (2020) 150:463–8. 10.1007/s11060-020-03465-0 32221783

[31] Galli J Morelli F Rigante M Paludetti G. Management of cerebrospinal fluid leak: the importance of multidisciplinary approach. Acta otorhinolaryngologica Italica: organo ufficiale della Societa italiana di otorinolaringologia e chirurgia cervico-facciale. Acta Otorhinolaryngol Italica. (2021) 41(Suppl. 1):S18–29. 10.14639/0392-100X-suppl.1-41-2021-02 34060517 PMC8172102

[32] Zhou ZY Wang XS Gong Y La Ali Musyafar O Yu JJ Huo G. Treatment with endoscopic transnasal resection of hypothalamic pilocytic astrocytomas: a single-center experience. BMC Surg. (2021) 21:103. 10.1186/s12893-021-01113-6 33632188 PMC7908641

[33] Zhou Y Hei Y Soto JM Jin T Jiang X Feng D. Clinical efficacy of the multilayered skull base reconstruction using *in situ* bone flap in endoscopic endonasal approach for craniopharyngioma. J Neurol Surg Part B, Skull Base. (2022) 83:e291–e7. 10.1055/s-0041-1726128 35832974 PMC9272260

[34] Schreckinger M Szerlip N Mittal S. Diabetes insipidus following resection of pituitary tumors. Clin Neurol Neurosurg. (2013) 115:121–6. 10.1016/j.clineuro.2012.08.009 22921808

[35] de Vries F Lobatto DJ Verstegen MJT van Furth WR Pereira AM Biermasz NR. Postoperative diabetes insipidus: how to define and grade this complication? Pituitary. (2021) 24:284–91. 10.1007/s11102-020-01083-7 32990908 PMC7966184

[36] Ajlan AM Abdulqader SB Achrol AS Aljamaan Y Feroze AH Katznelson L. Diabetes insipidus following endoscopic transsphenoidal surgery for pituitary adenoma. J Neurol Surg Part B, Skull Base. (2018) 79:117–22. 10.1055/s-0037-1604363 29868315 PMC5978855

[37] Araujo-Castro M Marino-Sanchez F Acitores Cancela A Garcia Fernandez A Garcia Duque S Rodriguez Berrocal V. Is it possible to predict the development of diabetes insipidus after pituitary surgery? Study of 241 endoscopic transsphenoidal pituitary surgeries. J Endocrinol Invest. (2021) 44:1457–64. 10.1007/s40618-020-01448-6 33043415

[38] Lin K Fan K Mu S Wang S. Change in cephalocaudal tumor cavity diameter after transsphenoidal surgery is a predictor of diabetes insipidus in pituitary adenoma. Eur J Med Res. (2022) 27:72. 10.1186/s40001-022-00700-4 35614499 PMC9131668

